# Chimpanzees breed with genetically dissimilar mates

**DOI:** 10.1098/rsos.160422

**Published:** 2017-01-11

**Authors:** Kara K. Walker, Rebecca S. Rudicell, Yingying Li, Beatrice H. Hahn, Emily Wroblewski, Anne E. Pusey

**Affiliations:** 1 Department of Evolutionary Anthropology, Duke University, Durham, NC, USA; 2 Sanofi, Cambridge, MA, USA; 3 Departments of Medicine and Microbiology, Perelman School of Medicine, University of Pennsylvania, Philadelphia, PA, USA; 4 Department of Structural Biology, Stanford University School of Medicine, Stanford, CA, USA

**Keywords:** inbreeding avoidance, mate choice, relatedness, kin recognition

## Abstract

Inbreeding adversely affects fitness, whereas heterozygosity often augments it. Therefore, mechanisms to avoid inbreeding and increase genetic distance between mates should be advantageous in species where adult relatives reside together. Here we investigate mate choice for genetic dissimilarity in chimpanzees, a species in which many females avoid inbreeding through dispersal, but where promiscuous mating and sexual coercion can limit choice when related adults reside together. We take advantage of incomplete female dispersal in Gombe National Park, Tanzania to compare mate choice for genetic dissimilarity among immigrant and natal females in two communities using pairwise relatedness measures in 135 genotyped chimpanzees. As expected, natal females were more related to adult males in their community than were immigrant females. However, among 62 breeding events, natal females were not more related to the sires of their offspring than immigrant females, despite four instances of close inbreeding. Moreover, females were generally less related to the sires of their offspring than to non-sires. These results demonstrate that chimpanzees may be capable of detecting relatedness and selecting mates on the basis of genetic distance.

## 1. Introduction

Inbreeding depression, a reduction of fitness in the offspring of closely related mates, is a widely documented phenomenon thought to result largely from the homozygous expression of recessive deleterious alleles [1–3]. Inbreeding can adversely affect fitness through numerous pathways, notably via early death or reproductive disadvantage [3]. In contrast, fitness is often positively correlated with increased heterozygosity, whether genome wide or at specific loci [4,5]. Selection should, therefore, favour the evolution of mechanisms to avoid inbreeding and promote mate selection of genetically different individuals, especially in species that live at high population densities or in social groups, and where the risk of encountering relatives is high [6,7].

Several potential mechanisms exist for inbreeding avoidance and mate choice. In many animals, the likelihood of close inbreeding is reduced via dispersal of individuals of one sex before breeding [8–11], but in some cases, close relatives continue to reside together as adults. In such instances, there is increasing evidence of behavioural avoidance of inbreeding where closely related pairs avoid mating altogether (e.g. elephants [12]; white-faced capuchins [13]) or tend only to mate when the likelihood of conception is low (e.g. macaques [14]). Kin recognition in such cases may be based on prior association and/or phenotype matching [6,15]. In other species, where relatives reside together as adults, mating among relatives sometimes occurs but offspring born to closely related parents are rare (e.g. blue tit [16]; hihi [17]). Post-copulatory mechanisms may account for this disparity, as illustrated by laboratory studies where females mate in short succession with a related and an unrelated male, but most offspring are fathered by the unrelated mate (e.g. field crickets [18]; agile antechinus [19]; house mice [20]).

A growing number of studies have also found evidence for mate choice for overall genetic dissimilarity in wild populations where the opportunity to encounter relatives is high. In highly philopatric banner-tailed kangaroo rats [21], Cunningham's skinks [22] and bushtail possums [23], breeding pairs are generally more distantly related than non-breeding pairs. Similarly, in greater sac-winged bats [24] and several bird species (e.g. reed buntings [25], red-backed fairy wrens [26], white-rumped swallows [27]), females breed with extra-pair or group males when those individuals are less related to them than their social mate or when their social mate is genetically similar. However, this phenomenon does not seem to be a general trend across species, even when the chance of inbreeding is high (e.g. Atlantic salmon [28], American pika [29], white-toothed shrews [30]), and recent meta-analyses in birds found no indication that relatedness differed between females and their social mate or their extra-pair mate [31,32]. Moreover, in some species, breeding pairs are more related than non-breeding pairs (e.g. pigeons [33]). While the mechanisms are unknown, choice for genetic dissimilarity probably depends on processes similar to those employed to avoid inbreeding.

In species in which females invest heavily in each offspring through gestation/incubation, lactation/provisioning and/or parental care, each offspring represents a significant proportion of a female's lifetime reproductive output such that the selection of a genetically different sire should be particularly important. If, in such species, the opportunity to mate with relatives is also elevated (e.g. through philopatry, promiscuity and/or sociality), there should be strong selection for multiple mechanisms that reduce close inbreeding and promote mating between unrelated pairs.

Chimpanzees (*Pan troglodytes*) have several features that are likely to favour the evolution of strong mate choice mechanisms for genetic dissimilarity. They have a very slow reproductive schedule, investing heavily in a single offspring at a time, and only giving birth approximately every 5 years [34]. Although most females disperse to other social groups before breeding, some remain in their natal group with their male relatives [35]. Long lifespans also result in males and some females residing as adults in the same social group as their opposite-sexed parents. Chimpanzees are highly promiscuous; during a female's sexual cycle, she mates multiply with most or all of the males in her community [36]. Preliminary evidence from both captive and wild populations suggests inbred offspring suffer high mortality [37,38]. Under these conditions, inbreeding should not be tolerated [7]. However, among eastern chimpanzees (*Pan troglodytes schweinfurthii*), males use sexual coercion to influence female mating patterns, probably restricting female choice [39–41]. The socioecology and mating patterns of chimpanzees thus put them at risk of conceiving with a close relative and potentially limit female choice for dissimilar mates. Therefore, mechanisms that reduce the chances of inbreeding and favour genetically dissimilar mates should be particularly important in this species.

Behavioural avoidance of mating with close relatives has been documented in chimpanzees but is more complete among some classes of relatives, identified by pedigree information, than others. Mother–son mating is rare across sites but has occasionally been observed [42–45], and the only offspring that has been identified as resulting from closely related parents in any study population came from a mother–son pairing [38]. Avoidance of mating between maternal siblings also occurs in some populations [42,45] but not others [46]. Such avoidance of maternal relatives is consistent with kin recognition by prior association [6]. The degree to which mating between paternal relatives occurs is less well known. In the only study to examine this, mating frequency between father–daughter dyads was surprisingly high, after accounting for male age and other known correlates of mating, and was primarily driven by male behaviour [47]. Daughters resisted the mating attempts of fathers at higher rates than those of unrelated males. There is some evidence for preferential interaction between males and their infant and juvenile offspring in this species [48,49], but the degree to which preferential interaction and/or association persists past juvenility is uncertain. Therefore, some form of phenotype matching may be involved in the identification of fathers in adulthood. In another study, dyadic relatedness calculated from microsatellite data was negatively related to mating frequency but mating did occur in most dyads [41]. Taken together, these patterns suggest that there is some recognition of relatedness which influences mating patterns in this species but that mating tactics may constrain choice for unrelated partners. As yet, the consequences of these patterns for the parentage of infants have not been examined. That is, the extent to which inbreeding is successfully avoided or genetic dissimilarity is favoured is not yet known.

Here we take advantage of differing demographic characteristics in two eastern chimpanzee communities in Gombe National Park, Tanzania to investigate patterns of mate selection and the factors influencing these by examining the parentage of 64 infants. The two communities (Kasekela and Mitumba) differ in size, number of adult males and the proportion of females that disperse. The larger Kasekela community is unusual in that about 50% of females breed in their natal community [50], whereas in Mitumba, 83% of females have dispersed, and the community currently contains only one natal female. First, we ask how effectively inbreeding is avoided by examining the incidence of moderate and close inbreeding based on known pedigree information. Second, we test our expectation based on sex-biased dispersal patterns that natal females are more closely related to resident males, and therefore at higher risk of inbreeding than immigrant females. Third, we examine the extent to which natal females are able to avoid inbreeding by testing whether they are more closely related than immigrant females are to the sires of their offspring. Last, we test the hypothesis that, because of the general advantages of heterozygosity, there will be overall mate choice for genetic dissimilarity whereby breeding dyads are less related than non-breeding dyads.

## 2. Methods

### 2.1. Study site

Gombe National Park, in western Tanzania, has an area of 35 km^2^ and contains three chimpanzee communities. The chimpanzees of the Kasekela and Mitumba communities have been habituated and observed almost daily since 1966 and 1995, respectively [51], and the dispersal patterns of all females born into or entering these communities are known. Individuals in the Kalande community are known genetically from faecal sampling [52]. All individuals surviving beyond 2 years of age and alive between 1995 and 2012 in Kasekela and 2004 and 2012 in Mitumba have been genotyped. Within these periods, the Kasekela community included 12–25 adult females (age 12+ years) and 10–12 adolescent and adult males (age 12+ years), and the Mitumba community included five to nine adult females and two to five adolescent and adult males.

Since the 1990s, the park has been isolated from other forested areas and is primarily surrounded by anthropogenic landscapes, potentially limiting dispersal opportunities [53]. However, healthy females occasionally disappear from the park shortly after sexual maturity and females of unknown provenance sometimes immigrate into communities in the park. In the last 5 years, two immigrant females have been determined to originate from outside the park; they were never observed in Mitumba or Kasekela and their genotypes did not match any known chimpanzee, including those genetically sampled in the Kalande community. A chimpanzee community of at least 20 individuals is known to reside approximately 12 km north of the park (D. Mjungu 2016, personal communication). Although this community is likely to be a potential source and destination for dispersing females, it is highly unlikely that offspring born within the park are sired by males outside the park, because extra-group paternities are rare in chimpanzees [38,54–57] and, in this case, would require significant travel across risky landscapes by within-park females.

### 2.2. Genotyping, paternity and relatedness

Included in this study were 153 genotyped chimpanzees from the Kasekela, Mitumba and Kalande communities [38,52,56,58]. Most individuals (98.6%) were typed at 8–11 microsatellite loci, whereas two individuals were typed at 4–5 loci (electronic supplementary material, figure S1*a*). More information on sample collection, DNA extraction and microsatellite genotyping (primarily from faeces) is provided in the electronic supplementary material.

Paternities for 37 offspring born into the Kasekela community had previously been reported (including one set of non-identical twins (GLI, GLD) whose paternity was tested individually) [38,52,56,58]. For this study, we repeated the analysis for these 37 paternities and we also report paternities for 28 new individuals born into the Kasekela and Mitumba communities. This provides a total sample size of 65 offspring with known paternities: 52 in the Kasekela community and 13 in the Mitumba community (electronic supplementary material, figure S2). All infants had known mothers that were also genotyped, and we had good sampling of candidate males, with genotypes for a mean of 97.2% of candidate males from within the community, and 91.6% of males between the two habituated communities (KK and MT; electronic supplementary material, figure S2). Ten is the youngest age documented for a chimpanzee sire in the wild [59], whereas 11 is the youngest age of any sire at Gombe (2015, unpublished data). All males at least 9 years of age at the time of conception that were resident in the mother's community (either Kasekela or Mitumba) were genotyped for 57 conceptions (87.7%), and all males were sampled from both communities for 41 (63.1%) conceptions (electronic supplementary material, figure S2). Fathers were first identified using the exclusion principle and confirmed with likelihood methods using the program Cervus [60]. Detailed information on the paternity assignment procedure is included in the electronic supplementary material.

#### 2.2.1. Pedigrees and inbreeding

We classified offspring as inbred if the father was related to the mother at the level of half-siblings (*R* = 0.25) or above based on known pedigrees. Using known pedigrees combined with genetic paternity information, we can detect any inbreeding between parent–offspring dyads, full siblings and maternal half-siblings for all genotyped offspring but can detect inbreeding between paternal siblings in only 53 of 65 offspring. Because extra-group paternity is rare in chimpanzees [38,54–57], we assumed that sires of offspring born to immigrant mothers were unrelated to the mother. Among 32 offspring born to natal mothers, 10 were born to parents where the father of both the mother and the sire was unknown, preventing us from determining if they were paternal siblings. Two mothers in Mitumba were present as adults at the time of habituation and their provenance is unknown (i.e. natal or immigrant); to be conservative, the offspring born to these mothers were also excluded from the determination of rates of inbreeding among paternal siblings (*n* = 2). A lack of generational depth in the pedigree prevented a systematic analysis of moderate inbreeding between other relatives related at the 0.25 level (i.e. grandmothers and grandsons, aunts and nephews, etc.) [61].

#### 2.2.2. Pairwise relatedness measures

We used Co-ancestry to calculate pairwise relatedness [62]. In this programme, allele frequencies were calculated once from the same set of microsatellite genotypes (8–11 loci) of 135 individuals from the Kasekela and Mitumba communities (electronic supplementary material, figure S1*c*). Bias can result from using the same markers to determine paternity and calculate relatedness [63–65]; however, our microsatellite data could not be split into two subsets as recommended, because the full set is necessary to determine paternity. Nonetheless, the high degree of polymorphism and heterozygosity of our markers reduces the potential for bias [63] (electronic supplementary material, figure S1*a*,*c*) and we found that when we calculated *R*-values based on different allele frequency subsets as recommended by Wang [65], values were highly correlated with the full dataset (see electronic supplementary material).

To facilitate comparison with previous work on genetic relatedness in chimpanzees [54,55,66], we used the Queller and Goodnight pairwise relatedness estimator [67] to compare relatedness between classes of dyads. We found that estimates of pairwise relatedness from this method correlated highly with kinship coefficients calculated between dyads of known parentage confirmed through paternity analysis and pedigree information (*n* = 70) using the KINSHIP2 package in R (see electronic supplementary material, table S1 and figures S3,S4) [68]. These estimates were also correlated with six other pairwise estimators calculated in Co-ancestry [62] (see electronic supplementary material, table S1) including the more recent triadic likelihood (TL) method [69], which allows for inbreeding, weights loci based on their informativeness and accounts for typing errors. We repeated our analysis using the TL method and found similar results (see electronic supplementary material, figures S5–S7).

### 2.3. Definitions and inclusion criteria

#### 2.3.1. Females and offspring

All females present in each community, aged 12 years or over and encountered in more than 10% of male focal follows (i.e. resident rather than peripheral community members [70]) were considered adults for relatedness analyses and were categorized as either natal (*n*_Kasekela_ = 15, *n*_Mitumba_ = 1) or immigrant (*n*_Kasekela_ = 20, *n*_Mitumba_ = 9) depending on their community of birth. Two females in Mitumba were of unknown provenance (they were present in the community when observations began) and were excluded from analyses in which females of different residence status were compared. Females that did not give birth (*n*_Kasekela_ = 8, *n*_Mitumba_ = 3) or have offspring genotyped (*n*_Kasekela_ = 3, *n*_Mitumba_ = 1) were excluded from analyses that required the use of breeding females. For the purposes of this study, the conception of twins was treated as a single event because they were sired by the same male (electronic supplementary material, figure S2). This leads to a total of 64 breeding events (51 in Kasekela and 13 in Mitumba).

#### 2.3.2. Males

All males 12 years or older and present in the community during the study period were considered adults for relatedness analyses (*n*_Kasekela_ = 24, *n*_Mitumba_ = 9). All males were born in the community in which they resided as adults except one (BE) that immigrated into Kasekela with a female assumed to be his sister at approximately 4 years of age [36]. The dominance rank of each male on the estimated date of conception of each infant [36,71] was given by his Elo score, calculated from the occurrence of submissive pant grunts [72]. In Mitumba, rank was only known for the alpha male, preventing a systematic analysis of rank and relatedness in this community.

### 2.4. Analyses of pairwise relatedness

#### 2.4.1. Female–male dyadic relatedness

To test for differences in the degree of genetic relatedness between natal and immigrant females with resident males, we calculated and compared average relatedness between all immigrant female–male dyads (*n* = 388) with average relatedness between all natal female–male dyads (*n* = 279) that overlapped as adults in the Kasekela community. This analysis was restricted to the Kasekela community, beause Mitumba had only one natal female (with no infant). Differences between natal female–male dyads and immigrant female–male dyads were tested for significance using a permutation procedure that compares the normalized difference between means from 10 000 random pairs of replicates from the pooled dataset using JMP v. 11 [73].

#### 2.4.2. Relatedness of natal and immigrant breeding dyads

Relatedness between immigrant mothers and sires of 30 offspring (19 in Kasekela and 11 in Mitumba) was compared with relatedness between natal mothers and sires of 32 offspring (all in Kasekela) in two ways: (i) including both communities and (ii) restricting analysis to just Kasekela since, as above, Mitumba had only one natal female (with no infant). Differences between groups were tested using the same permutation procedure explained above.

#### 2.4.3. Relatedness of breeding and non-breeding dyads

Relatedness between breeding and non-breeding dyads was examined by comparing the relatedness between the mother and sire of an infant (breeding dyads) with the relatedness between the mother and each other adult male present in the community at the conception of her infant (non-breeding dyads) [36,71]. We tested each community separately, using 50 offspring in the Kasekela community and 11 offspring in the Mitumba community. One offspring born to a transient mother in Kasekela (KAR) and two offspring born in Mitumba prior to 2004 (APL and LAM) were excluded from this analysis because not all candidate sires were genotyped. Differences between groups were first compared using the same permutation procedure explained above. To determine if any observed difference was due to female residence status, we also compared breeding and non-breeding dyads among natal and immigrant females separately.

Male rank and age influences the probability of paternity [56]. Therefore, to be confident that selection of breeding partners was influenced by genetic distance, in addition to other known factors, we also ran a linear mixed model using the lme4 package [74] in R [75] controlling for male rank and age. Relatedness was the outcome variable and dyad type (breeding or non-breeding), male rank and male age were included as fixed effects, and male and female ID were both included as random effects. To investigate if breeding patterns varied by community, we ran a second linear mixed model where relatedness was the outcome variable, and dyad type, community and the interaction term community × dyad type were included as fixed effects. Male ID and Female ID were also included as random effects.

## 3. Results

### 3.1. Paternity and inbreeding

The fathers identified for all 37 offspring reported previously were confirmed here, with one exception (DIA). Typing at additional microsatellite loci resolved paternity between two maternal half-brothers for DIA, which we update here (electronic supplementary material, figure S2). Detailed information on paternity likelihood is included in the electronic supplementary materials. All 65 offspring, including 28 for which paternity is newly reported here, were sired by within-community males except for one (KAR), whose mother (TT) resided primarily in the southern Kalande community but conceived during a visit to Kasekela (table 1). KAR was also determined to be inbred, a product of breeding between maternal siblings. TT emigrated before her maternal brother was born and the conception occurred when she revisited her natal community after a sudden decrease in the adult male population in her transfer community (see the electronic supplementary material) [52].

**Table 1.** Paternities and related demographic information for 65 offspring.

community	offspring	mother	sire	mother's residence status	new paternity	inbred	status in 2016 (age at death in years)
Kasekela	CN	CD	WL	natal			
Kasekela	COC	CD	FD	natal			
Kasekela	DIA	DL	FO	natal	Y^d^		
Kasekela	DUK	DL	TN	natal	Y		
Kasekela	FO	FF	WL	natal			
Kasekela	**FI**	FF	FR	natal		mother–son	dead (0.9)
Kasekela	FE	FF	EV	natal			
Kasekela	FLI	FF	KS	natal			
Kasekela	FU	FN	SL	natal			
Kasekela	FND	FN	SL	natal			
Kasekela	FAM	FN	SL	natal			
Kasekela	FAD	FN	WL	natal			
Kasekela	FFT	FN	WL	natal	Y		
Kasekela	**GGL**	GA	FO	natal	Y	paternal sibs	alive
Kasekela	GAb1	GA	SL	natal	Y		
Kasekela	GAb2	GA	AO	natal	Y		
Kasekela	GLA	GLD	FU	natal	Y		
Kasekela	GLIb1	GLI	SL	natal	Y		
Kasekela	GD	GM	AL	natal			
Kasekela	GA	GM	WL	natal			
Kasekela	GLI/GLD^a^	GM	FR	natal			
Kasekela	GIM	GM	TB	natal			
Kasekela	**GIZ**	GM	FE	natal	Y	paternal sibs^c^	dead (6.3)
Kasekela	SR	SA	BE	natal			
Kasekela	SN	SA	AO	natal			
Kasekela	SAM	SA	FR	natal			
Kasekela	SIR	SA	AO	natal			
Kasekela	SAF	SI	AO	natal	Y		
Kasekela	SHA	SR	WL	natal			
Kasekela	TOM	TG	KS	natal			
Kasekela	TAB	TG	FE	natal	Y		
Kasekela	**KAR**	TT	TN	natal^b^	Y	maternal sibs	dead (approx. 2^b^)
Kasekela	BRZ	BAH	KS	immigrant			
Kasekela	BAS	BAH	TN	immigrant	Y		
Kasekela	ERI	EZA	KS	immigrant			
Kasekela	EZAb1	EZA	TN	immigrant	Y		
Kasekela	IPO	IMA	WL	immigrant	Y		
Kasekela	JK	JF	AL	Immigrant			
Kasekela	KEA	KP	WL	immigrant			
Kasekela	MAM	MAK	GL	immigrant			
Kasekela	NYO	NUR	FU	immigrant	Y		
Kasekela	TG	PI	GB	immigrant			
Kasekela	TN	PI	FR	immigrant			
Kasekela	TZN	PI	FR	immigrant			
Kasekela	SI	SW	WL	immigrant			
Kasekela	SDB	SW	FR	immigrant			
Kasekela	TOF	TTA	SL	immigrant			
Kasekela	ZS	TZ	FR	immigrant			
Kasekela	ZEL	TZ	KS	immigrant			
Kasekela	ZIN	TZ	GL	immigrant			
Kasekela	YAM	YD	WL	immigrant			
Mitumba	APL	AP	VIN	immigrant	Y		
Mitumba	AND	AP	EDG	immigrant	Y		
Mitumba	ARI	AP	RUD	immigrant	Y		
Mitumba	MAY	DB	RUD	immigrant	Y		
Mitumba	FLW	FS	RUD	immigrant	Y		
Mitumba	FAL	FS	EDG	immigrant	Y		
Mitumba	FED	FS	EDG	immigrant	Y		
Mitumba	KOM	KON	RUD	immigrant	Y		
Mitumba	LAM	LUC	VIN	immigrant	Y		
Mitumba	LTT	LUC	EDG	immigrant	Y		
Mitumba	MIS	MGA	EDG	immigrant	Y		
Mitumba	EDE	EVA	VIN	unknown	Y		
Mitumba	LOS	LOR	RUD	unknown	Y		

^a^Twins, confirmed to have the same father, treated as one conception.

^b^TT is transient, born in KK, currently resides in Kalande with occasional visits to KK such that age at death for KAR is approximate.

^c^Strong but not conclusive evidence that offspring is inbred.

^d^Paternity previously reported as FE.

Of the 28 new infants, two were the products of inbreeding (*R* ≥ 0.25 as determined from pedigree data) and strong evidence points to a third case (table 1). KAR was the offspring of maternal half-siblings (as discussed above); GGL and probably GIZ (see the electronic supplementary material) were the offspring of paternal half-siblings.

Offspring conceived by close relatives (*R* = 0.5; *n* = 1) accounted for 1.5% of all genotyped infants and 3% of those born to natal females. Offspring conceived by half-siblings (*R* = 0.25; *n* = 3) account for 5% of all genotyped infants and 12% of those born to natal females. We currently lack depth of pedigree to identify most offspring born to other relative classes at the 0.25 level; however, in the instances where such relationships were known, no inbreeding was detected.

Three of the four inbred infants died prior to attaining sexual maturity; two died around or prior to 2 years (FI and KAR) and the third (GIZ) died at 6 years. Importantly, none of these infants, nor their mothers, were positive for simian immunodeficiency virus (SIVcpz) infection, which is known to increase infant mortality [76].

### 3.2. Female–male dyadic relatedness

**Figure 1.**
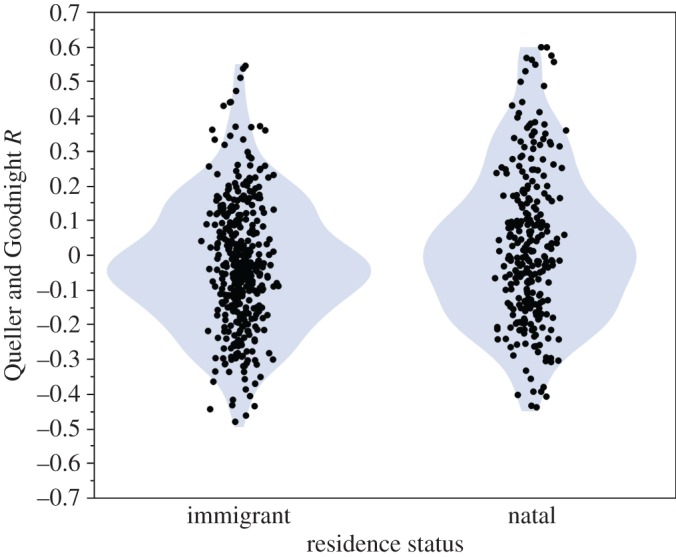
Relatedness between immigrant female–male dyads and natal female–male dyads in Kasekela. Natal female–male dyads are more closely related than are immigrant female–male dyads (*p* < 0.001).

In Kasekela, adult female–adult male dyads (*n* = 667) had a mean relatedness of −0.009 (s.d. = 0.198). In accordance with relatedness patterns expected from sex-biased dispersal, natal females were more closely related to the males of their community (*n* = 279 dyads, mean *R* = 0.022, s.d. = 0.216) than immigrant females were (*n* = 388 dyads, mean *R* = −0.031, s.d. = 0.183; *p* < 0.001; figure 1).

### 3.3. Relatedness of natal and immigrant breeding dyads

Despite the fact that natal females were more closely related to resident males, natal females were not more closely related to the sires of their offspring (*n* = 32 dyads, mean *R* = −0.052) than were immigrant females (*n* = 30 dyads, mean *R* = −0.063; *p* = 0.81), including data from both communities. There was also no significant difference when analysis was restricted to dyads from the Kasekela community (natal breeding dyads; *n* = 32 dyads, mean *R* = −0.052; immigrant breeding dyads: *n* = 19 dyads, mean *R* = −0.101; *p* = 0.35).

### 3.4. Relatedness of breeding and non-breeding dyads

**Figure 2.**
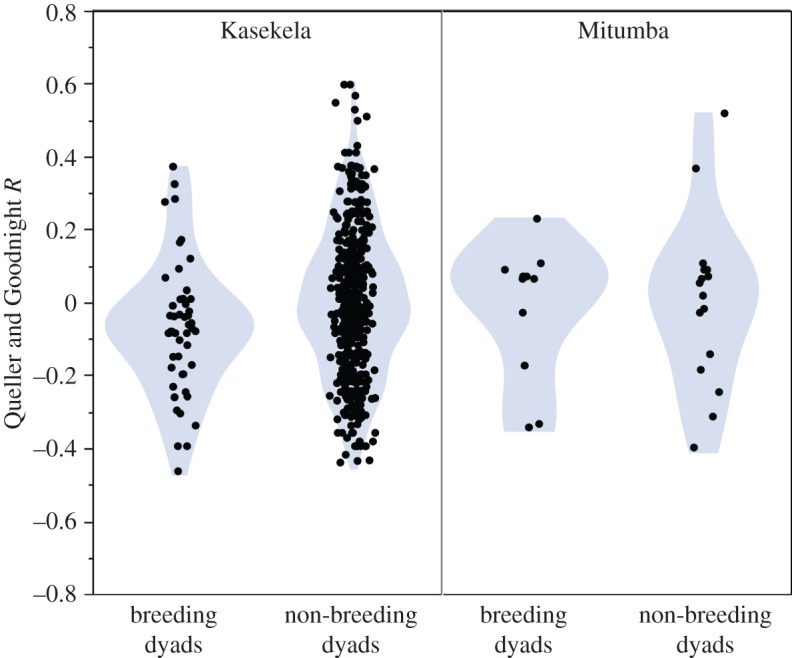
Relatedness between breeding and non-breeding dyads in Kasekela and Mitumba. In Kasekela, breeding dyads are less related than non-breeding dyads (*p* = 0.017).

**Figure 3.**
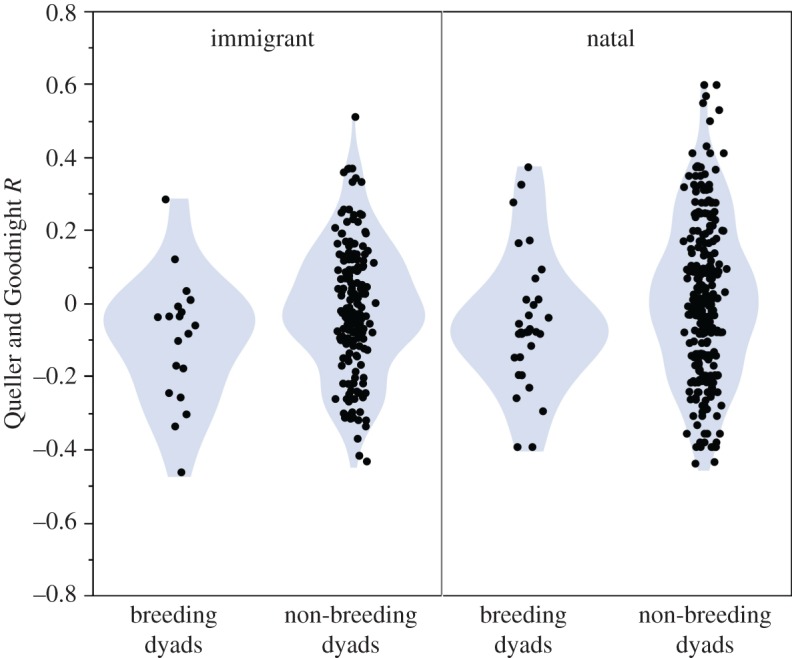
Relatedness between breeding and non-breeding dyads in Kasekela by residence status. Among immigrant females, breeding dyads are less related than non-breeding dyads (*p* < 0.01) and among natal females, there was a trend in the same direction (*p* = 0.10).

In the Kasekela community, breeding dyads including both natal and immigrant females (*n* = 50 dyads, mean *R* = −0.072) were significantly more distantly related than non-breeding dyads (*n* = 486 dyads, mean *R* = 0.000, *p* = 0.01; figure 2). This difference remained significant in a model that included the rank and age of the male (*β* = 0.07, *t* = 2.397, *p* = 0.017). A similar difference was found in the Mitumba community but did not reach significance (breeding dyads: *n* = 11 dyads, mean *R* = −0.017; non-breeding dyads: *n* = 16 dyads, mean *R* = 0.003; *p* = 0.81; figure 2). That the pattern generally applies to both communities is supported by the linear mixed model results, which shows that while the dyad effect remains significant when including both communities (*β* = 0.07, *t* = 2.40, *p* = 0.017), there is no significant effect of community (*β* = 0.04, *t* = 0.55, *p* = 0.58) or in the community × dyad type interaction (*β* = −0.05, *t* = −0.65, *p* = 0.51). To determine if the bias towards unrelated breeding partners was a general pattern or if it was driven by natal females avoiding close relatives, we compared dyads for immigrant and natal females in Kasekela separately (figure 3). For immigrant females, breeding dyads (*n* = 19, mean *R* = −0.101) were still significantly more distantly related than non-breeding dyads (*n* = 181 dyads, mean *R* = −0.018, *p* < 0.01) and, for natal females, there was a trend in the same direction (breeding dyads: *n* = 31, mean *R* = −0.049; non-breeding dyads: *n* = 305, mean *R* = 0.011, *p* = 0.10).

## 4. Discussion

Our results suggest that, as expected for a slowly reproducing species in which females invest heavily in each offspring and sometimes reside with close relatives, several mechanisms promote inbreeding avoidance and mate choice in chimpanzees. First, sex-biased dispersal reduced the relatedness between breeding adults in the community: immigrant females were more distantly related to the males in the community than were females that did not disperse. Second, although they often resided with close male relatives, natal females were not more closely related to the sires of their offspring than were immigrant females. Third, females in the Kasekela community were more distantly related to the sires of their offspring than to the other resident males. These latter two patterns provide evidence for the existence of mechanisms to detect genetic similarity among individuals and promote outbreeding.

Nonetheless, these systems are not perfect and we identified four cases of inbreeding; one between closely related parents and three between moderately related parents. Three of these occurred between relatives residing together. One resulted from a rare forced son–mother mating [38], whereas two occurred between females that did not disperse from their natal Kasekela community and their male paternal siblings. The fourth case occurred between maternal siblings under unusual circumstances in which the parents never resided together. We may have underestimated the prevalence of inbreeding in the population, because most infants that die prior to age two are not genotyped (19 of 23 offspring in Kasekela and 2 of 2 offspring in Mitumba); however, 15 of these infants were born to immigrant mothers and were unlikely to be inbred. Furthermore, we were only able to measure the extent of inbreeding between half-siblings and parents/offspring because, owing to the long lifespan of chimpanzees, our pedigree was not deep enough to accurately assess rates of other forms of inbreeding [61]. In the cases where we could identify grandparents/grandchildren and aunts/uncles/nieces/nephews, we found no evidence of inbreeding. Although the sample size is small, the outcome of the four cases of inbreeding is suggestive of inbreeding depression. Fifty per cent of inbred offspring died in the first 2 years and 75% died by age six, compared with an overall average infant mortality of 29% in the first 2 years and 40% in the first 6 years [77].

In support of selection for genetically dissimilar mates, we observed that breeding pairs were more distantly related than non-breeding pairs. Importantly, this result was not driven by natal females avoiding close relatives as breeding partners. Rather, we found a general bias among all females, including immigrant females, for unrelated mates. These patterns suggest that chimpanzees are able to detect degrees of genetic relatedness and breed with more distantly related individuals and do not simply avoid relatives with whom they have a history of association. While we found a strong effect of choice for genetic dissimilarity in Kasekela, the effect was diminished in the smaller Mitumba dataset, though when modelled together, no community difference was found. Nevertheless, it is possible that demographic differences between communities may influence patterns of mate choice. In small groups, more effective mate guarding by the alpha male may constrain female choice, whereas larger groups may provide more scope for alternative mating strategies such as consortships [56,78] and future work, with a larger sample, should clarify these effects. Mate choice for genetic dissimilarity is likely to confer fitness benefits on the resultant offspring and infant survival is, thus, expected to vary by the degree of relatedness between the parents. Here, we document high infant mortality in moderate and close inbreeding events, but future work should examine fitness effects across all degrees of relatedness.

An alternative explanation for the difference in relatedness between breeding and non-breeding pairs is that more genetically similar pairs do breed but that offspring born to these pairs suffer high infant mortality in the first 2 years and are, thus, generally not included in the genotyped sample. However, this explanation is unlikely, given that selection for dissimilar mates is more robust among immigrants, the class of females that faces the lowest risk of inbreeding. Moreover, the majority of non-genotyped infants were born to immigrant mothers and unlikely to be inbred. Nevertheless, when possible, efforts to genotype young infants should be undertaken to confirm the origin of this effect.

Our data indicate that genetic relatedness influences mate choice but cannot distinguish between female or male effort in such choice nor do they speak to the mechanisms involved in the detection of degrees of relatedness, both issues that merit further investigation. Choice of good genes is particularly important for mammalian females because of their investment in gestation and lactation [34,79,80]. Accordingly, female chimpanzees frequently do not respond to mating invitations from maternal male relatives and will often resist if a relative persists in a mating attempt [36,42,45]. However, males also contribute to inbreeding avoidance and, once past sexual maturity, rarely mate with their mothers and infrequently pursue maternal sisters [36,42,43,45].

Identifying the mechanisms that animals use to determine relatedness is an active area of research. In chimpanzees, prior association with the mother during the juvenile period is a likely mechanism for detection of maternal relatives [6,15,45] and it is striking that the only case of inbreeding between maternal siblings occurred in a pair that had no contact prior to adulthood. Pre-copulatory detection of paternal relatives or general genetic difference is more likely to depend on phenotype matching on the basis of such cues as odour similarity [81–84], or physical resemblance to known relatives [85,86]. In addition, because mating does occur between relatives, but infants rarely result, post-copulatory sperm selection or cryptic female choice like that detected in agile antechinuses and house mice and suspected to occur in mandrills may also be important [19,20,87].

In conclusion, we provide evidence that chimpanzees breed with genetically dissimilar mates and that inbreeding is uncommon even where opposite sex adult relatives reside together. Chimpanzees are probably sensitive to genetic distance in choosing a mate, although post-copulatory processes may also contribute to observed patterns of outbreeding. Such mechanisms should optimize genetic diversity in the resultant offspring and increase their fitness.

## Supplementary Material

Electronic Supplementary Material - This single file contains detailed information on the paternity assignment procedure, including genbank numbers and a summary of paternity analyses. It also contains information on and checks of potential biases in our calculation of pairwise relatedness measures and compares our generated pairwise relatedness values to Hamilton's R for individuals of known pedigree. It contains supplementary information on two offspring; one of whom's determination of inbreeding was less precise than usual and one who was conceived in unusual circumstances. Finally, we also duplicate our results presented in the main paper using triadic likelihood pairwise relatedness values

## Data accessibility

Genotypes and pairwise relatedness data are available from the Dryad Digital Repository: http://dx.doi.org/10.5061/dryad.d7g4c [88].
