# Supplementary material for: Chimpanzees breed with genetically dissimilar mates
Source: R Soc Open Sci. 2017 Jan 11;4(1):160422. doi: 10.1098/rsos.160422 (PMC5319312; doi:10.1098/rsos.160422)
Supplement: Electronic Supplementary Material - This single file contains detailed information on the paternity assignment procedure, including genbank numbers and a summary of paternity analyses. It also contains information on and checks of potential biases in our calculation of pairwise relatedness measures  [file rsos160422supp1.docx]

**SUPPORTING MATERIALS**

**Chimpanzees breed with genetically dissimilar mates.**

KARA K. WALKER, REBECCA S. RUDICELL, YINGYING LI, BEATRICE H. HAHN, EMILY WROBLEWSKI, & ANNE E. PUSEY

**Methods**

***Sample collection and storage***

Fecal samples were collected from individuals within the habituated Kasekela and Mitumba communities immediately after observing defecation into an equal volume of feces:RNAlater (Ambion) (~20-25ml each). Feces were generally collected near nest sites for the Kalande chimpanzees. In the event of an animal’s death, tissue samples were collected into RNAlater (Ambion) during necropsy. Samples were frozen (-20°C) on the same day of collection in the Kasekela community, whereas samples from the Mitumba and Kalande communities were stored at ambient temperature before being transported to the field lab in Kasekela and frozen (typically within a week). Samples were then transported by air at ambient temperature from Gombe to the Hahn laboratory in the United States, where samples were then stored frozen at -80°C (total transit time from Gombe to the Hahn lab is about one week).

***DNA extraction, sample identification and microsatellite genotyping***

We extracted DNA for this study from fecal samples using the protocol described by Wroblewski et al. (2009, 2015) and the QIAamp DNA Stool Mini Kit (Qiagen). Tissue samples were extracted using the DNeasy Blood and Tissue Kit (Qiagen). For the newly determined paternities, DNA was extracted from fecal samples for 23 individuals and from tissue samples from five individuals whose bodies were recovered after death (GAb1, GAb2, GLIb1, EZAb1, and AND). At least two independent samples per individual, whenever available, were used for DNA extraction and microsatellite PCR genotyping. Alternatively, at least two independent PCR reactions were done from a single sample in the rare event that only one was available. Of 153 genotyped individuals from the three communities (Figure S1A), seven were genotyped from tissue collected during necropsy. For the remaining 146 individuals typed from fecal samples, three infants from Kasekela and 10 individuals sampled in Kalande had only a single fecal sample available. Eight individuals from Kasekela only had published genotypes (no samples) available from Constable et al. (2001). Thus, of 138 individuals genotyped since Constable et al. (2001), 125 (90.8%) have been genotyped from more than a single sample.

The visual identification made by sample collectors of the chimpanzee sample donor was confirmed using several means (further described in the respective references): 1.) PCR-based sex determination (Wroblewski et al. 2009, Sullivan et al. 1993), 2.) mitochondrial hypervariable D loop haplotyping (confirming that samples from offspring and their known mothers had the same haplotype) (Wroblewski et al. 2009, Liu et al. 2010), and 3.) genotyping at 8-11 microsatellite loci (again confirming that offspring and their known mothers shared at least one allele at every locus) (Constable et al. 2001, Wroblewski et al. 2009, Rudicell et al. 2010, Gilby et al. 2012, this study).

***Paternity***

Fathers were first identified using the exclusion principle and confirmed with the likelihood methods using the program Cervus (Kalinowski et al. 2007). In Cervus, allele frequencies were calculated using all 153 genotyped individuals and 10,000 offspring were simulated. We conservatively included all genotyped males (62) as candidate fathers, regardless of community residence or known dates of birth or death, but we also conservatively simulated 90% sampling of candidate males. We also simulated 1% error in genotyping and likelihood calculations. Simulations enabled confidence levels to be assigned to identified fathers at the relaxed confidence level of 80% or the strict confidence level of 95%. Those 62 genotyped males were included as candidate fathers in the Cervus paternity analysis for all 65 offspring, with only the offspring being excluded by Cervus as a candidate male in the analysis of their own paternity (i.e. the offspring couldn’t be considered as a candidate father to itself). In the three cases in which 8-11 loci, along with life history and demographic information, were not sufficient to resolve paternity, genotyping at 9-10 additional loci was done for offspring and the unresolved candidate males (Figures S1B, S2).


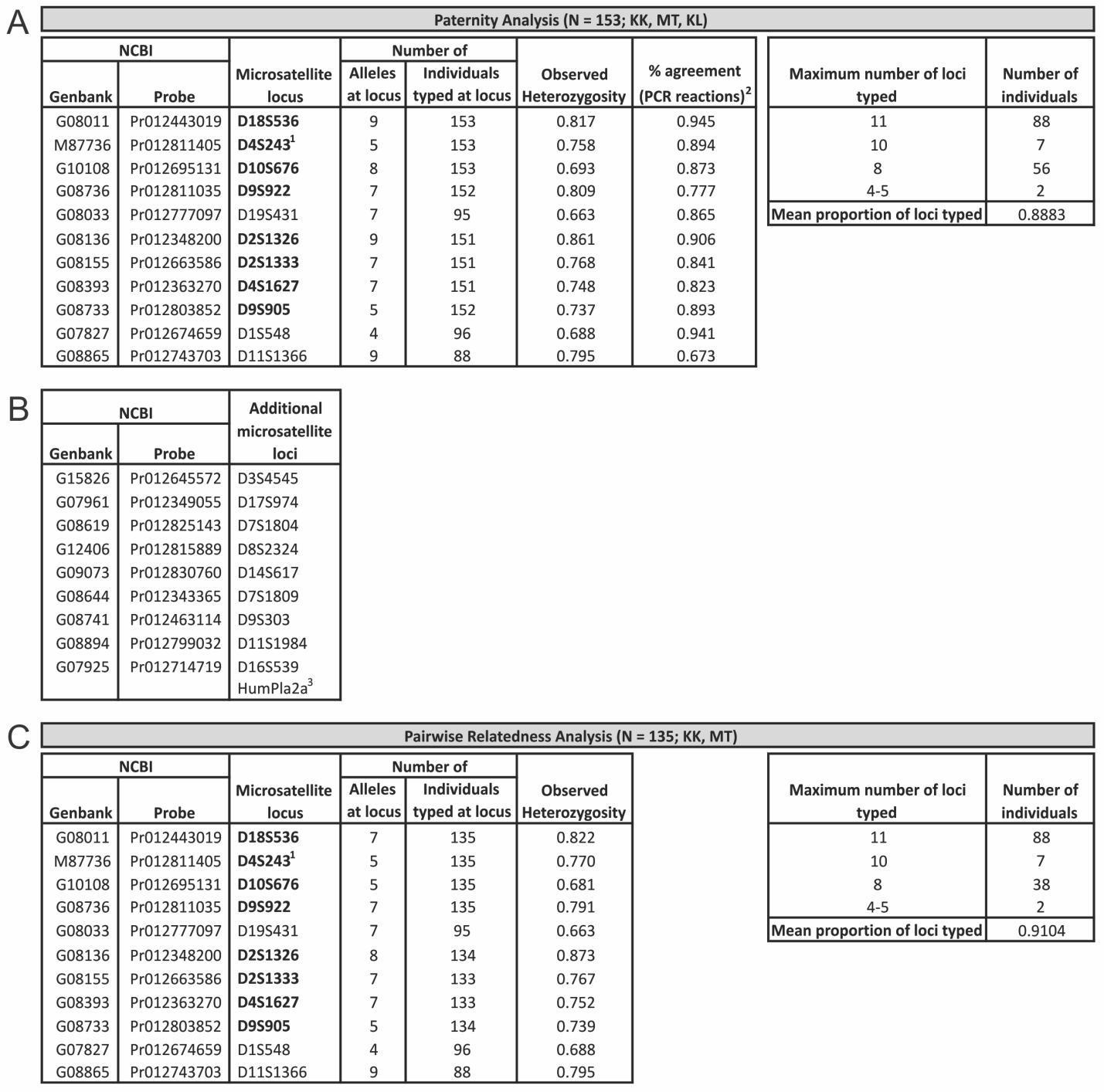


**Figure S1. Summary of genetic data for paternity and relatedness analyses.**

**(A)** N denotes the number of chimpanzees included from the Kasekela (KK), Mitumba (MT), and Kalande (KL) communities. To the left is a summary of the microsatellite loci analyzed while to the right is a summary of the genotyping of the individual chimpanzees included. Included are NCBI Genbank and Probe database accession numbers. In bold are the eight loci at which individuals are standardly genotyped. ^1^Hudson et al. ^2^Percent agreement between results of PCR genotyping reactions (a least two performed) for a subset of 84 KK and MT individuals genotyped for Wroblewski et al. (2009) and this study. **(B)** Given is a list of the additional microsatellite loci genotyped to resolve paternity when the standard 8-11 loci (A) were insufficient. Included are NCBI Genbank and Probe database accession numbers. ^3^Ghobrial et al. **(C)** N denotes the number of chimpanzees included from the Kasekela (KK) and Mitumba (MT) communities. To the left is a summary of the microsatellite loci analyzed while to the right is a summary of the genotyping of the individual chimpanzees included. In bold are the eight loci at which individuals are standardly genotyped. Included are NCBI Genbank and Probe database accession numbers. ^1^Hudson et al.


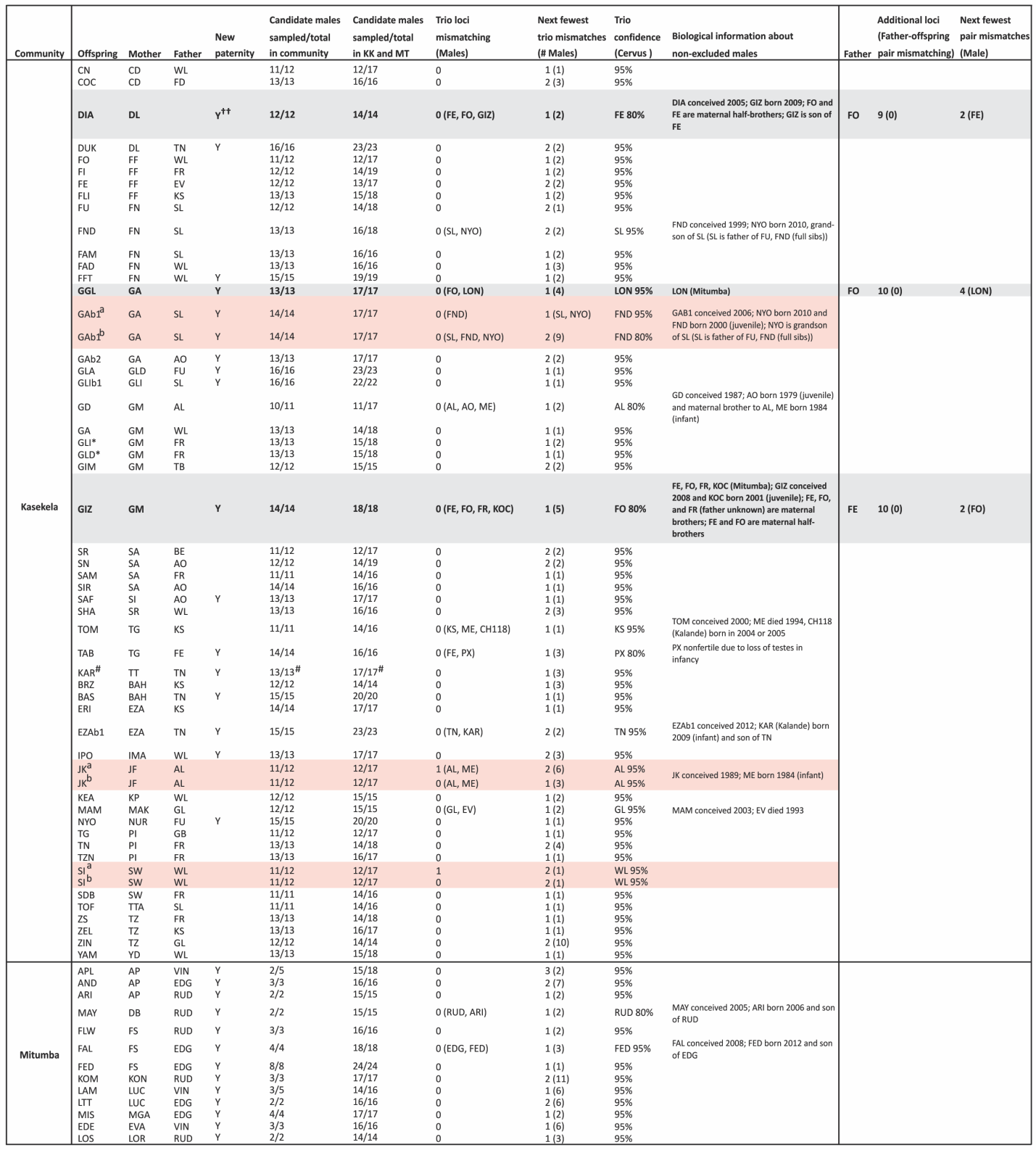


**Figure S2. Summary of paternity analyses.** Numbers of candidate males sampled/total include males nine years of age or older. Three paternity assignments requiring additional loci to compare between the father-offspring pair are in bold font and highlighted in gray. Highlighted in light orange are three paternities in which the assigned father had a single locus mismatching between the genotypes of the offspring-mother-father trio^a^, however this likely was the result of genotyping error, and removal of the mismatching locus from the analysis resulted in zero mismatches^b^. *GLI and GLD are non-identical twins. ^#^TT is transient, born in KK, currently resides in Kalande with occasional visits to KK. Therefore candidate males in Kasekela (KK) and Mitumba (MT) are estimated based on the approximate conception date of KAR. ††Paternity previously reported as FE (Wroblewski et al. 2009), typing at 9 additional loci, for a total of 20, shows father is FO (maternal brother to FE).

***Pairwise Relatedness Measures***

To explore potential biases in our calculation of R values, we calculated R values based on allele frequencies calculated from three different subsets of individuals. Given the duration of the study, we calculated allele frequencies using: 1) those individuals alive at the beginning of the study (1995) and 2) alive at the end of the study (2012). Third, given that the inclusion of relatives in the calculations of allele frequencies can bias the resulting R values Wang (2014), we used the extensive pedigree to identify a subset of 20 unrelated individuals in the 2012 population. With all three alternative calculations of allele frequencies, the resulting R values were highly correlated with those calculated from the total data set of 135 individuals sampled in Kasekela and Mitumba (correlations with the total data: R^2^ = 0.980 (start, 1995), = 0.995 (end, 2012), = 0.985 (unrelated)), and therefore did not change our results. Therefore, we used the total genetic data set to not discard information regarding allele frequencies.

**Table S1**: R^2^ values between the kinship coefficient calculated from individuals of known parentage in KINSHIP2 and six pairwise relatedness estimators calculated in the program Co-ancestry. All values are significant at the p <0.001 level (n = 591 dyads).

|  | TL | DL | Wang | Lynch & Li | Lynch & Ritland | Ritland | Queller & Goodnight | **Kinship Coefficient** |
| --- | --- | --- | --- | --- | --- | --- | --- | --- |
| TL |  | 0.951 | 0.733 | 0.730 | 0.783 | 0.606 | 0.759 | **0.500** |
| DL | 0.951 |  | 0.771 | 0.761 | 0.789 | 0.598 | 0.784 | **0.474** |
| Wang | 0.733 | 0.771 |  | 0.987 | 0.720 | 0.520 | 0.892 | **0.336** |
| Lynch & Li | 0.730 | 0.761 | 0.987 |  | 0.710 | 0.511 | 0.909 | **0.328** |
| Lynch & Ritland | 0.783 | 0.789 | 0.720 | 0.710 |  | 0.850 | 0.803 | **0.401** |
| Ritland | 0.606 | 0.598 | 0.520 | 0.511 | 0.850 |  | 0.621 | **0.334** |
| Queller & Goodnight | 0.759 | 0.784 | 0.892 | 0.909 | 0.803 | 0.621 |  | **0.342** |
| **Kinship Coefficient** | **0.500** | **0.474** | **0.336** | **0.328** | **0.401** | **0.334** | **0.342** |  |


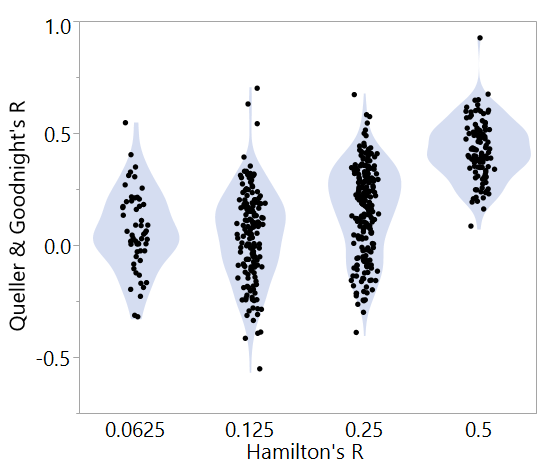


**Figure S3**: Queller & Goodnight R for individuals of known pedigree (Hamilton’s R).


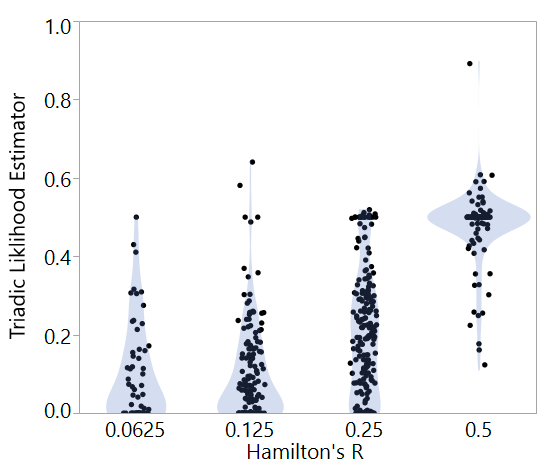


**Figure S4:** Triadic Likelihood Estimator (TL) for individuals of known pedigree (Hamilton’s R).

**Special Cases**

**KAR** KAR’s mother is TT and he was sired by TN. TT was born to PI in Kasekela in 1984 and immigrated to Mitumba in 1993 where her presence was confirmed via daily observations. She was last seen in Mitumba in 1998. She was later identified through genetic analysis of fecal samples to be residing in the southern Kalande community. Beginning in 2006 and continuing to the present, she has made periodic visits to her natal Kasekela community (ranging from single day visits to those lasting several weeks) following the community decline of Kalande in the early 2000s (Rudicell et al. 2010). She was observed in December of 2010 with a young infant estimated to have been born in July; later named Karibu (KAR) and determined conclusively to have been fathered by TN. TN was born to PI in 1994, after TT had immigrated to Mitumba and the two never resided together, excepting TT’s occasional visits. KAR was last seen with TT in October, 2012 and is presumed dead as TT has since been observed without KAR and now has a new infant.

**GIZ** Evidence strongly suggests GIZ’s parents FE & GM were paternal siblings but this could not be conclusively determined. GIZ was born to GM and FE was conclusively determined to be his father. FE’s father was conclusively determined to be EV. GM, the mother of GIZ, was conceived in the Kasekela community in 1970 and when genotyping began in the 1990s GM’s mother was dead and only one candidate sire, EV, was still alive. However EV was identified as the father through likelihood analysis with 80% confidence under the most conservative parameters (Wroblewski et al., 2009).

**RESULTS**

***Paternity & Demographic Information***

Using the exclusion principle, 51 infants had a single male identified as the father (0 loci mismatching between the father-mother-offspring trio), which were also confirmed by likelihood-based methods in Cervus at the 95% confidence level (Figure S2). Eleven offspring had two or three non-excluded males, but a father could be identified in eight of these cases based on exclusionary biological information available for the other males, such as known dates of birth (and ages) or death, given the habituation and detailed long-term study of the Mitumba and Kasekela communities and the long-term monitoring and observation of the Kalande community. In five of these eight cases, non-excluded males were also known relatives of each other. Nonetheless, in all but one (TAB) of the eight cases, Cervus still identified the living and/or viable (reproductive-aged) male at the 80% or 95% confidence level. In the three cases in which a father could not be confidently identified using biological information (DIA, GGL, GIZ), genotyping the non-excluded males at additional loci then excluded all males except one, the assigned father. Finally, three offspring (GAb1, JK, and SI) each had a single mismatch between the identified father-mother-offspring trio. This was likely the result of genotyping error since excluding the mismatched locus from the analysis resulted in a single, living and viable male with zero mismatches within the trio. Thus, overall, paternity identified by exclusion agreed with paternity determined by likelihood-based methods (Cervus) for 61 out of 65 offspring (93.8%).

***Relatedness Analyses Using TL***

Analyses presented in the main paper are repeated here using the Triadic Likelihood Estimator (TL; Wang 2007) rather than Queller & Goodnight’s pairwise relatedness estimator (Queller & Goodnight, 1989) (Figures S5-S7). The findings remain the same regardless of measure.

Figure S5: Violin plot of relatedness between immigrant female-male dyads (n = 388, mean R = 0.054, SD = 0.091) and natal female-male dyads in Kasekela (n = 279, mean R = 0.092, SD = 0.154; p < 0.001).

Mitumba

Kasekela*

B)

A)


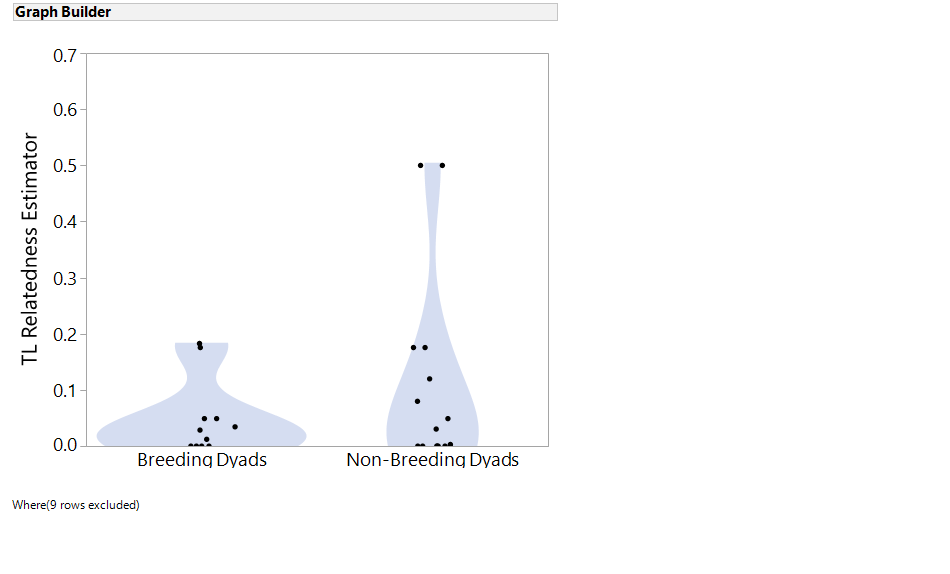

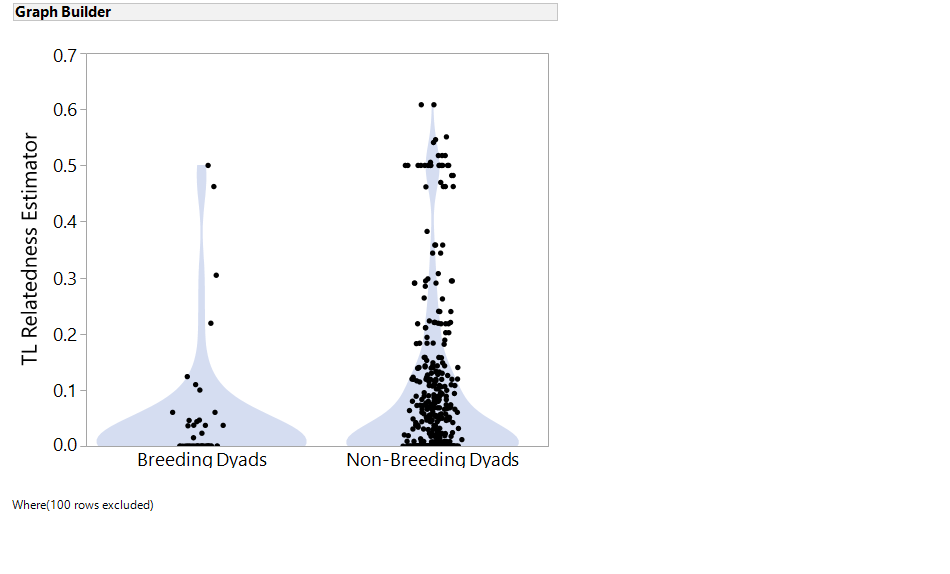


**Figure S6:** Violin plot of relatedness between A) breeding (n = 50, mean R = 0.045, SD = 0.107) and non-breeding dyads (n = 486, mean R = 0.079, SD = 0.006) in Kasekela (p = 0.04) and B) breeding (n = 11, mean R = 0.049, SD = 0.020) and non-breeding dyads (n = 16, mean R = 0.102, SD = 0.167) in Mitumba (p = 0.26).

**Figures S7:** Relatedness between immigrant breeding (n = 19, mean R = 0.022, SD = 0.010) and non-breeding dyads (n = 181, mean R = 0.056, SD = 0.088; p = 0.005) and relatedness between natal breeding (n = 31, mean R = 0.059, SD = 0.131) and non-breeding dyads (n = 305, mean R = 0.093, SD = 153; p = 0.18) in Kasekela.

**References**

Constable, J.L., Ashley, M. V., Goodall, J., Pusey, A.E., 2001. Noninvasive paternity assignment in Gombe chimpanzees. *Molecular Ecology*. 10, 1279–1300.

Ghobrial, L., Lankester, F., Kiyang, J. A., Akih, A. E., De Vries, S., Fotso, R., ... & Gonder, M. K. 2010. Tracing the origins of rescued chimpanzees reveals widespread chimpanzee hunting in Cameroon. *BMC ecology*. 10, 1.

Gilby, I.C., Brent, L.J.N., Wroblewski, E.E., Rudicell, R.S., Hahn, B.H., Goodall, J., Pusey, A.E., 2013. Fitness benefits of coalitionary aggression in male chimpanzees. *Behavioral Ecology and Sociobiology*. 67, 373–381.

Hudson, T.J., Engelstein, M., Lee, M.K., Ho, E.C., Rubenfield, M.J., Adams, C.P., ... & Dracopoli, N.C. 1992. Isolation and chromosomal assignment of 100 highly informative human simple sequence repeat polymorphisms. *Genomics*. 13, 622-629.

Kalinowski, S.T., Taper, M.L., Marshall, T.C., 2007. Revising how the computer program CERVUS

accommodates genotyping error increases success in paternity assignment. *Molecular Ecology*. 16, 1099-1106.

Liu W, Li Y, Learn GH, Rudicell RS, Robertson JD, et al. (2010) Origin of the human malaria parasite Plasmodium falciparum in gorillas. Nature 467: 420-425.

Queller, D., Goodnight, K., 1989. Estimating relatedness using genetic markers. Evolution. 43, 258–275.

Rudicell, R.S., Holland Jones, J., Wroblewski, E.E., Learn, G.H., Li, Y., Robertson, J.D., Greengrass, E., Grossmann, F., Kamenya, S., Pintea, L., et al., 2010. Impact of simian immunodeficiency virus infection on chimpanzee population dynamics. *PLoS Pathogens*. 6, e1001116.

Sullivan KM, Mannucci A, Kimpton CP, Gill P. 1993. A rapid and quantitative DNA sex test: fluorescence- based PCR analysis of XY homologous gene amelogenin. Biotechniques 15: 636-638, 640-631.

Wang, J., 2007. Triadic IBD coefficients and applications to estimating pairwise relatedness. *Genetical Research*. 89, 135-153.

Wang, J.L., 2014. Marker-based estimates of relatedness and inbreeding coefficients: an assessment of current methods. *Journal of Evolutionary Biology.* 27, 518-530.

Wroblewski, E.E., Murray, C.M., Keele, B.F., Schumacher-Stankey, J.C., Hahn, B.H., Pusey, A.E., 2009. Male dominance rank and reproductive success in chimpanzees, *Pan troglodytes schweinfurthii*. *Animal Behaviour*. 77, 873–885.

Wroblewski, E. E., Norman, P. J., Guethlein, L. A., Rudicell, R. S., Ramirez, M. A., Li, Y., ... & Parham, P. 2015. Signature patterns of MHC diversity in three Gombe communities of wild chimpanzees reflect fitness in reproduction and immune defense against SIVcpz. *PLoS Biology*, 13, e1002144.
